# Seasonal Variation in Fungi in Beach Sand in Summertime: Stintino (Italy)

**DOI:** 10.3390/ijerph20237134

**Published:** 2023-12-01

**Authors:** Massimo Deligios, Vittorio Mazzarello, Maura Fiamma, Aleksandra Barac, Lorenzo Diana, Marco Ferrari, Manuela Murgia, Bianca Paglietti, Salvatore Rubino

**Affiliations:** 1 Department of Biomedical Sciences, University of Sassari, 07100 Sassari, Italy; deligios@uniss.it (M.D.); vmazza@uniss.it (V.M.); lorenzodiana18@gmail.com (L.D.); manuelamurgia@hotmail.com (M.M.); biancap@uniss.it (B.P.); 2 Laboratorio Analisi, Ospedale “San Francesco”, ASSL Nuoro, 08100 Sardinia, Italy; fiammamaura@gmail.com; 3 Clinic for Infectious and Tropical Diseases, Clinical Centre of Serbia, Faculty of Medicine, University of Belgrade, 11000 Belgrade, Serbia; aleksandrabarac85@gmail.com

**Keywords:** fungi, skin, microbiota, beach, sand

## Abstract

Background: The goal of this study was to monitor the microbial biodiversity in beach sand that is heavily visited by tourists during the summer, and to determinate whether the high presence of bathers (around 5000 per day) can modify sand microbial composition. Methods: Between 2016 and 2020, 150 sand samples were collected from nine different points at La Pelosa beach in Sardinia, Italy. Non-culturing methods were used; DNA extraction and meta-barcode sequencing were performed. All samples were analyzed with sequencing methods for 16S and ITS sequences. Results: Fungal genera differ on the three beaches and in the winter/summer zones. The ITS sequence showed the most common presence of *Candida* during summer and *Paradendryphiella* in the winter. The greatest diversity was found in the dune during winter, while in other parts of the beach, there are differences between bacteria and fungi, particularly in the wash zone during the winter, with high diversity for 16S sequences but low diversity for ITS sequences. Conclusions: It appears reasonable that the sands, even on non-urban beaches, should be included in health monitoring programs in addition to the waters, and that access to them should be regulated by limiting the number of bathers with the aim of reducing the presence of pathogenic fungal species.

## 1. Introduction

The microbiological quality of bathing water is one of the most important indicators of a beach’s environmental and health quality available to users. In fact, most countries have water monitoring programs in place to protect swimmers’ health. In reality, while all of the skin is in contact with the water during a bath, almost all bathers spend more time in contact with the sand during a day at the beach. A wide range of microorganisms can be found in beach sand. Sand grains, in fact, act as a natural filter, creating a wide variety of protected microhabitats rich in nutrients that favor the survival, and possibly growth, of microorganisms known as micropsammon, which include viruses, bacteria, fungi, and protozoa. This was confirmed by analyzing the sand from a large number of recreational beaches in many countries around the world, from which numerous bacterial and fungal species were isolated [1,2,3,4,5,6,7,8,9,10,11,12,13,14,15,16,17,18,19,20,21,22]. Some of these are potentially pathogenic and, at high concentrations, can be harmful to humans’ health [18].

Pathogenic bacteria are typically the result of anthropogenic wastewater pollution, and their presence correlates with a variety of factors. In fact, various indicators of fecal contamination, including enterococci, *Escherichia coli*, coliform bacteria, *Clostridium perfringens*, and staphylococci, have been isolated, particularly in the swash zones of beaches, including freshwater ones, which are very crowded and integrated within large cities [1,4,10,11,13,15,16,20]. There is a well-established cause-and-effect relationship between the fecal pollution of recreational waters and outbreaks of gastroenteritis [23], ocular, nasal, ear, and throat symptoms among swimmers [24,25]. There have been few studies that link the levels of microbial contaminants in sand to risks to human health. One study discovered that gastrointestinal symptoms would develop if all of the bacteria on the fingertip were ingested via hand-to-mouth transfer of *E. coli* present in sand [26]. Another study discovered a link between fecal-derived microbes in sand and gastroenteritis in beachgoers who dug or buried themselves in the sand [27].

Other potentially pathogenic fungi, such as yeasts (*Candida* sp.) [1,9,18], molds (*Aspergillus* sp.) [9], and dermatophytes [3], have been discovered in the sand of some beaches, in the presence or absence of bacterial indicators of fecal contamination. However, these new findings do not tell us whether their origin is a simple contamination of the sand by skin fungi or a result of environmental factors. Fungi can be found in the soil as well as in the sea. They can reach the beach from the ground via wind or rainwater run-off from adjacent impermeable surfaces such as parking lots and sidewalks [28]. Their presence in the sea can be attributed to wastewater, indicating that they are of intestinal origin [3,19,29,30], or to their role in the decomposition of organic substrates, nutrient recycling, and hydrocarbon biodegradation [31,32]. Despite their low concentration, fungi perform the same functions of decomposition and nutrient recycling with a fertilizing action in the soil [33]. Some studies suspect that fungal contamination of the sand is caused by contact with human skin; however, this hypothesis was developed indirectly by comparing beaches with different anthropic pressures [3,7,8,14,17,18,20].

Beaches in Sardinia are very popular, so it is necessary to assess their healthiness. Because they are primarily non-urban, far from important residential areas and industrial waste, surrounded by Mediterranean scrub with reduced decomposition phenomena, and have a low presence of seabirds and domestic animals, they can be used as a model to demonstrate the possible contribution of sunbathers alone to the microbiological quality of sand.

As a result, the goal of this study was to monitor the microbial biodiversity in the sand of a beach in northern Sardinia that is heavily visited by tourists during the summer for five years using methods independent of culture, with the goal of determining whether a high presence of bathers can modify sand microbial composition by adding potentially pathogenic species.

## 2. Materials and Methods

### Sampling Site and Method

The annual monitoring beach, known as “La Pelosa”, is about 4 km from Stintino, a small town in northern Sardinia with a population of about 1500 people. It was frequented by a large number of bathers at the start of the study, with an average of 5000 daily presences in the summer. Access to the beach is via wooden walkways, which are a long distance from a paved road separated by sand dunes. La Pelosa beach is a cuspate foreland with a surface area of 7080 m^2^ and a slope of no more than 2% throughout the study period. It is a coastal deposit with medium and fine granulometry, with the majority of its sedimentary contribution coming from the submerged marine sector. The sandy cusp is surrounded by a dune system formed by sand blown by the wind, which primarily comes from the sea (with a prevailing north–northwest direction) and has settled on the rocky slope behind it.

**Figure 1:**
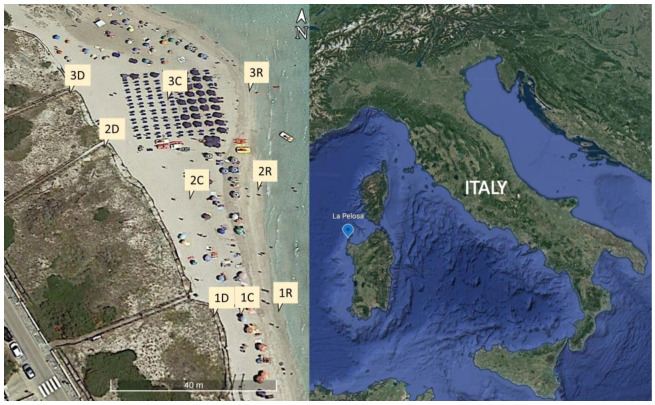
Map showing the location of sampling points on the beach “La Pelosa”, Stintino, Italy. Google Earth, earth.google.com/web/. The letter indicates the place on the beach, C: center; R: shore; D: dune. The number is the position: south (1), middle (2), and north (3).

Between 2016 and 2020, 150 sand samples were collected from 9 different points. Three points were in the wash zone (R) under the influence of the sea, three in the dry sand of the beach center (C) where bathers usually live, and three more in the dunes (Ds) on the beach’s outskirts (Figure 1). On non-high tide days, the samples were collected around 11 a.m. Sand was collected at 5 cm from the surface from each GPS-located point and stored in a 50 mL sterile test tube. The date, time, GPS point, location, and sampling temperature were all recorded for each sample. Monitoring was carried out during the winter (October–February) and summer (May–September) seasons. The samples were immediately taken to the laboratory for processing after they were collected.

Beginning in 2019, events occurred that significantly reduced the number of bathers as a result of municipal regulations and COVID-19. The most important regulations imposed by the Municipality of Stintino were a maximum of 1012 bathers per day, no smoking, and no pets. These new facts have enabled a different analysis of the data accumulated over 5 years, dividing it into two groups: pre-municipal regulation period (2016–2018) and post-municipal regulation period (2019–2020).

## 3. Methods and Statistical Analysis

### 3.1. DNA Extraction and Meta-Barcode Sequencing

A DNeasy PowerMax Soil Kit (QIAGEN, Inc., Valencia, CA, USA) was used to extract DNA. Samples from the same beach location (D, C, and R) were pooled and processed with minor modifications according to the manufacturer’s instructions. To obtain a greater amount of DNA, 15 g of sand was extracted rather than 10 g. A Qubit fluorimeter was used to analyze DNA using a QubitTM dsDNA HS Assay Kit (Life Technologies, Carlsbad, CA, USA). The DNA was sent to BMR Genomics (Padua, Italy) for quality control and barcode sequencing for bacterial (16S) and fungal (ITS) identification. In brief, primers V3–V4 and ITS2 were used to amplify 16S rRNA and the internal transcribed spacer (ITS), respectively. Illumina MiSeq Paired End sequences (2 × 300 bp) were used for sequencing. Unsuitable samples for sequencing were discarded. Sequences were received and downloaded for further examination.

### 3.2. Bioinformatics Analysis

All samples’ 16S or ITS sequences were merged and imported into the Qiime2 package. Sequences were filtered, merged, and chimeras were removed. Using the R v0.6.5 microecho package, high-quality sequences were processed for downstream analyses such as taxonomy (in terms of ASVs, Amplicon Sequence Variants), alpha and beta diversity, and assays of differential abundance. The sequences were compared with the Silva Release 132 and Unite 8.0 classifiers (ref.) for bacteria and fungi, respectively, and represented in terms of taxonomic abundance in the three parts of the beach (shore = R, center = C, and dune = D) during the summer tourist season (between May and September = S) and winter (non-tourist) (between October and March = W). The Shannon index was used to calculate alpha diversity using a boxplot, whereas beta diversity was evaluated using unweighted UniFrac and represented as a principal coordinate analysis (PCoA) plot. For bacteria and fungi, diversity was calculated separately. Finally, a differential abundance test was performed between the center before and after the limitations using the LEfSe method [34].

## 4. Results

### 4.1. Bacterial and Fungal Taxonomy

Bacterial genera differ on the three beaches and in the winter/summer zones (Figure 1, left). During the summer, the most common genera in the center of the beach are Gramella, Salimicrobium, and Salegentibacter, with Woeseia also present. Throughout the year, Adhaeribacter, Blastocatella, and Flavisilibacter were identified in the dune. In the Wash zone, Woeseia was always present, whereas Marinobacter was only found during the summer. The ITS sequence-identified genera (Figure 1, right) show the presence of Candida in the center during summer and Paradendryphiella in the winter. Stemphylium, Peziza, Fusarium, and Alternaria were found in all dune samples, with the exception of Vishniacozyma and Aspergillus, which were found primarily in the summer. Homalogastra is the dominant genus found in the wash zone.

### 4.2. Alpha and Beta Diversity

**Figure 2:**
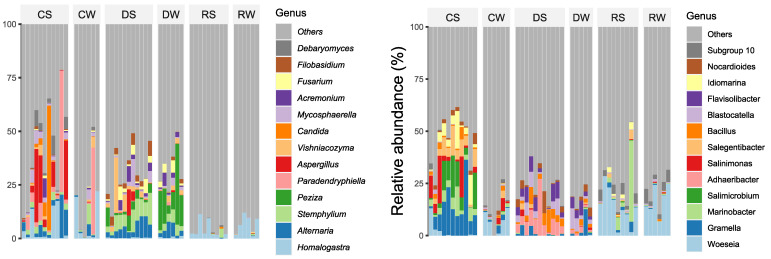
Taxonomy of 16S (**left**) and ITS (**right**) sequences. Each sample is labeled as follows: The first letter indicates the place on the beach, C: center; R: shore; D: dune. The second letter is the season, S: summer tourist season (between May and September); W: winter non-tourist season (between October and March).

**Figure 3:**
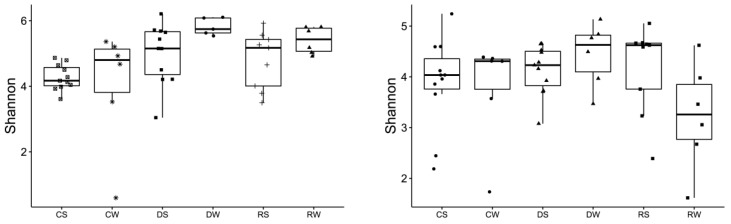
Shannon index of 16S and ITS sequences. Each sample is labeled as follows: C: center; R: shore; D: dune; S: summer tourist season (between May and September); W: winter non-tourist season (between October and March).

**Figure 4:**
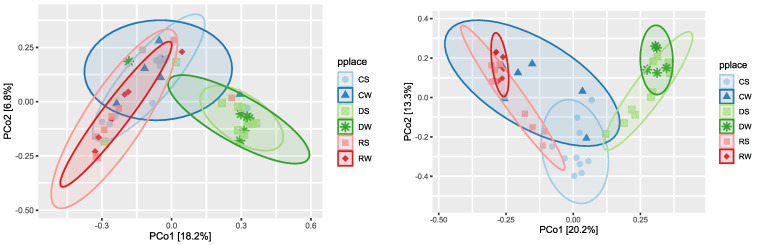
Beta diversity of 16S (**left**) and ITS (**right**) as unweighted Unifrac. Each sample is labeled as follows: C: center; R: shore; D: dune; S: summer tourist season (between May and September); W: winter non-tourist season (between October and March).

The greatest diversity was found in the dune during winter, as measured using the Shannon index in the 16S and ITS sequences (Figure 2, left and right, respectively). In other parts of the beach, there are differences between bacteria and fungi, particularly in the wash zone during the winter, with high diversity for 16S sequences but low diversity for ITS sequences. Unweighted Unifrac beta diversity was calculated and visualized with a PCoA plot (Figure 3, 16S left, ITS right). Bacteria and fungi clustered separately in the dune while the center and the shore overlap, but with a small separation in the bacteria in the center during summer (CS) (Figure 4, 16S left, ITS right).

### 4.3. Effect of Limiting the Number of Visitors to the Beach

**Figure 5:**
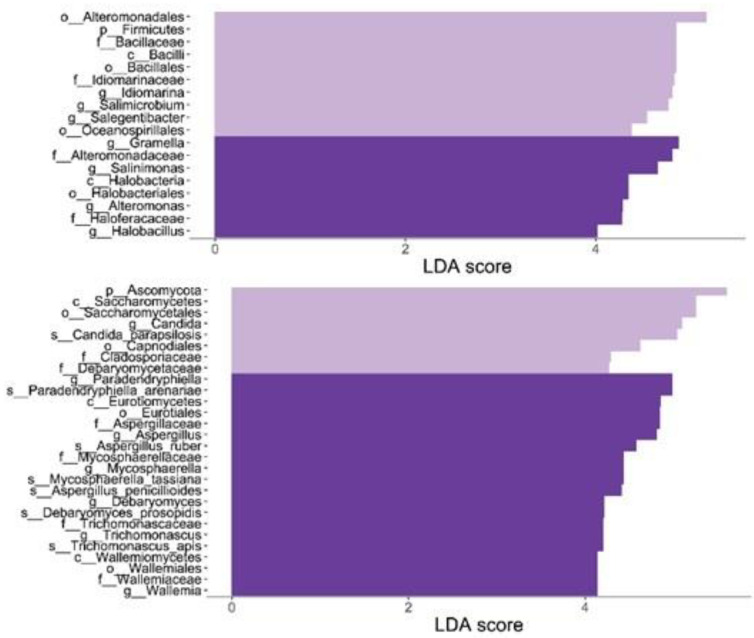
Taxa differentially abundant in the center during summer. pre- vs. post-limitations are displayed in light and dark violet, respectively.

During the summer, the differential abundance test with the LEfSe method was used at the center to distinguish between pre- and post-limitation groups (Figure 5; AC: pre-limitations, BC: post-limitations). Before the beach restrictions, the bacteria were significantly differentiated and belonged to the genera Idiomarina, Salimicrobium, Salegentibacter, and other higher ranks (AC). Gramella, Salinimonas, Alteromonas, Halobacillus, and other genera were identified after the restrictions (BC) (Figure 5, top). Prior to the bans, mushrooms, yeasts, and molds were discovered on the beach. The Candida parapsilosis species is the most common pathogenic yeast (AC). We discovered several taxa after settlement, including Aspergillus penicillioides and A. ruber (BC) (Figure 5, bottom). Table 1 depicts an examination of the species present in the skin microbiome in the literature, as well as the average percentage values between periods of high and low anthropic pressure due to restrictive municipal regulations.

## 5. Discussion

Coastal tourism is Sardinia’s first economic activity, having supplied the traditional products of sun, sand, and sea for decades, attracting tourists from all over the world who visit Sardinian beaches every day for several hours, including lunch hours, during the summer. We now know that sand, due to its structure, promotes the survival and possibly growth of microorganisms, and that some species found in it are potentially pathogenic and can pose a health risk at high concentrations [18]. As a result, conducting studies and performing microbiological analyses on beach sand are critical not only for detecting potential sources of microbial pollution, but also for correctly assessing probable risk situations and understanding how to prevent them, as well as understanding their potential role and implications in human health. For these reasons, a study was conducted from 2016 to 2020 to monitor all the microorganisms present in a recreational beach in Sardinia during the winter and summer seasons using the metagenomics technique.

The overall analysis of the data obtained revealed an annual consistency of microorganisms, both 16s and ITS, of environmental, marine, or coastal origin, with different biological profiles in the three areas studied, and with greater biodiversity in the dunes. In terms of human pathogens, no fecal indicators, *S. aureus*, or pathogenic dermatophytes were found in any of the samples examined. We only found potentially pathogenic fungi of human origin in summer samples taken from the dry sand of the central beach, which is the most affected by human presence. In fact, pathogenic skin yeasts of the *Candida* genus (21% of total fungi), *Malassezia*, *Saccharomyces*, and *Rhodotorula*, as well as traces of molds of the *Aspergillus* genus, were isolated during the summer months of the years without regulations (2016–2018). *Candida parapsilosis* was the most common *Candida* species isolated (19% of the total fungi), followed by *C. tropicalis*, *C. orthopsilosis*, and *C. albicans*. *Malassezia restricta*, *Malassezia globosa*, *Malassezia arunalokei*, *Saccharomyces cerevisiae*, and *Rhodotorula mucilaginosa* were the other non-*Candida* species isolated (Table 1).

All of these forms are found in the skin microbiota (Table 1), particularly in the feet [35], where they are thought to originate. Because these microorganisms are found on the skin, they are likely to tolerate high salinity and thus can survive in dry sand during the summer [18]. In vitro studies have shown that *Candida* and dermatophytes can survive for a few weeks even when salinity and temperature levels exceed those of their natural habitat [36]. Along with these, we discovered traces of other yeasts that have not yet been isolated on human skin, such as *C. quercitrusa*, *C. solani*, and *C. sanyaensis*, only in the center of the beach in the summer. These are environmental yeasts found in the soil, but their presence in the summer suggests that they are spread by tourists via their clothing or beach accessories. As a result, they could be the result of national/international travel affecting soil yeast communities by transferring species and genotypes from one country to another, which should be investigated further [37].

Again, in the summer, there was a clear reduction in yeasts of human origin in the center of the beach over the years with restrictive municipal regulations (2019–2020). In fact, the presence of only *C. parapsilopsis* and *C. albicans* resulted in a significant decrease in the percentage of the genus *Candida* (0.1% of the total fungi). During all of the winter months studied (2016–2020), there was a complete disappearance of pathogenic fungi in all three sampling sites, with only the *Rodotorula* and *Malassezia restricta* species present in traces (average 0.06%). The identification of the *Candida* species discovered on La Pelosa’s non-urban beach is consistent with other studies conducted on beaches around the world [1,3,7,8,15,18,19,20,21,22]. *Candida albicans* [1,3,7,8,15,18,19,22], *C. tropicalis* [7,18,19,21,22], and *C. parapsilopsis* [8,19,21] were the most common yeasts identified in these studies. However, all of these studies were conducted on urban beaches in metropolitan contexts with high organic pollution of wastewater, where a significant positive correlation was also demonstrated in various cases between yeasts and fecal coliforms present in the sand [21,22]. With our findings, we can confirm that *Candida* sp., which is also found on non-urban beaches such as La Pelosa, can be of cutaneous origin as a result of high anthropic pressure, as evidenced by the fact that their presence tends to disappear when the number of bathers is significantly reduced.

*Malassezia* sp. was rarely isolated from sea sand in previous studies [7], and in our study, three of these species are present in low concentrations only during the summer, with the exception of *M. restricta*, which is present in the sand all year. Until recently, it was assumed that Malassezia sp. evolved to inhabit the skin of warm-blooded mammals, from which 18 species have been isolated, with *M. restricta* being the most prevalent on human skin, particularly on the scalp [18]. They have also been isolated in other contexts in recent years, and it is thought that *M. globosa* and *M. restricta* are cosmopolitan [38]. Identical *M. restricta* DNA sequences have been found in a variety of habitats, including deep sea sediments [39], hydrothermal vents [40], corals [40], lobster larvae [41], eel japonica intestine and muscle tissue [42], Antarctic soils [43], soil nematode exoskeletons [44], and various plant roots [45]. It is also not surprising that *Malassezia* sequences are common in human habitation [46], where human skin contributes significantly to house dust. We believe that the presence of the genus *Malassezia restricta* on La Pelosa beach is of marine origin, as it has been found all year, whereas *M. globosa* and *M. arunalokei* are of human origin. *Malassezia* yeasts are thought to cause Pityriasis versicolor, *Malassezia*-related folliculitis, seborrheic dermatitis, and dandruff, but it is unclear whether these are contagious or can be transmitted by sand [47].

*Rhodotorula* spp., particularly *R. mucilaginosa*, have been discovered on heavily used urban and non-urban marine beaches [2,8,18,19,48]. *Rhodotorula mullacinosa* is widely distributed in nature, and we believe it is of environmental origin because it is present in the sand of La Pelosa all year. They have also been found in the sand of various beaches, including those in Italy [5,14,48,49], and in some cases associated with the presence of *Penicillium* sp. In the summer, we only found *Aspergillus* sp., whereas *Penicillium* sp. was isolated only in the dunes all year and in the center of the beach only in winter. Despite the fact that both are common contaminants in marine soils [50], the presence of *Aspergillus* in the summer suggests that it is of human origin, whereas *Penicillium* is of environmental origin.

*Saccharomyces cerevisiae* has been found on other Algerian beaches [48] and is only present during the summer; we believe it is of human origin. In contrast to other sites, we found no dermatophytes of the genera *Microsporum, Epidermophyton*, or *Trichophyton*. Several authors isolated these on sand samples, confirming their presence on various densely populated beaches only during the bathing season [8,17,20,22,51,52,53,54,55]. However, dermatophytosis epidemiology shows that this species is becoming less prevalent as an infectious agent [56], which could explain why we did not find it in La Pelosa sand.

Pathogens found on La Pelosa beach have been linked to numerous skin and systemic infections, primarily in immunocompromised patients and with an increasing frequency [32,57,58,59,60]. Furthermore, some *Aspergillus* species and *Malasseziae* species contain numerous cell wall components, such as chitin, glucans, mannans, mannoproteins, and galactomannan, which can cause irritation or hypersensitivity in susceptible individuals [8,9,61]. On the other hand, it is important to take into consideration that the weather is different between summer and winter, so the differences in the fungi species could be also due to weather effects and not only from beach visitors and bathers. A limitation of the study is that culture-based methods were not use for comparison, which could be very important for the design of the future studies.

In numerous works, the pathogens present on La Pelosa beach have often caused skin and systemic infections mainly in immunocompromised patients and with an increasing incidence [32,57,58,59,60]. Furthermore, some species of *Aspergillus* sp. and *Malasseziae* sp. contain many cell wall components, including chitin, glucans, mannans, mannoproteins and galactomannan, which may cause irritation or hypersensitivity phenomena in predisposed individuals [8,9,61].

## 6. Conclusions

Our findings would support the human origin of some pathogenic fungi found in the dry sand of Stintino’s La Pelosa beach during the summer. They are not marine in nature because they do not lack a swash zone, nor do they come from the ground because they are absent in the dunes behind the beach and in winter. Furthermore, the surrounding flora is composed of evergreen plants that decompose slowly. This study is significant because it confirmed that a large number of bathers alter the biodiversity of the sand with the appearance of fungi found in human microbiota, whereas a small number reduces their presence to a minimum. In our case, we went from having one person per square meter to having one every 7 m^2^. This resulted in a significant reduction in the number of pathogens. Among these, *C. parapsilosis* is the most important yeast in percentage terms, and it could be considered a parameter that can be used to evaluate whether a beach quota has had the desired effects or to detect any changes in the microbial population over time, allowing appropriate action. We agree with other studies that recommend examining dry sand for monitoring fungi because they can survive in a saline sandy environment longer than enteric bacteria, retaining their virulence and potential to cause diseases. As a result, it appears reasonable that the sands, even on non-urban beaches, should be included in monitoring programs in addition to the waters, and that access to them, particularly on overcrowded beaches, should be regulated by limiting the number of bathers.

## Figures and Tables

**Table 1:** The most common species commonly isolated from the skin microbiota. Each sample is labeled as follows: R: shore; C: center; D: dune; Summer: tourist season (between May and September); Winter: non-tourist season (between October and March).

		Summer						Winter					
Species	Localization (Skin)	C 2016/18	C 2019/20	R 2016/18	R 2019/20	D 2016/18	D 2019/20	C 2016/18	C 2019/20	R 2016/18	R 2019/20	D 2016/18	D 2019/20
*Candida parapsilosis*	Foot	18.8%	0.10%	0.42%									
*Candida tropicalis*	Nails	1.33%	0.01%										
*Candida orthopsilosis*	Newborn skin	0.80%											
*Candida albicans*	Foot	0.14%	0.02%										
*Candida boidinii*	Back	0.04%											
*Saccharomyces cerevisiae*	Foot	0.39%	0.26%		0.03%								
*Rhodotorula mucilaginosa*	Foot	0.14%	0.16%	0.01%		0.01%		0.02%	0.08%				
*Malassezia restricta*	Foot, back	0.32%	0.03%	0.02%	0.09%			0.09%		0.04%	0.01%	0.04%	0.01%
*Malassezia arunalokei*	Seborrheic zone	0.04%											
*Malassezia globosa*	Back	0.03%	0.02%										
*Aspergillus penicillioides*	Head (sebum)	0.48%	5.05%										
Fungi of probable human origin:		22.51%	5.64%	0.45%	0.12%	0.01%		0.11%	0.08%	0.04%	0.01%	0.04%	0.01%
	*Candida*	21.1%	0.13%	0.42%									
	*Malassezia*	0.40%	0.05%	0.02%	0.09%			0.09%		0.04%	0.01%	0.04%	0.01%
	Other yeasts	0.53%											
	Molds	0.48%	5.05%										

## Data Availability

The data presented in this study are available on request from the corresponding author. The data are not publicly available due to privacy reasons.
